# Design and Characterization of a New pVII Combinatorial Phage Display Peptide Library for Protease Substrate Mining Using Factor VII Activating Protease (FSAP) as Model

**DOI:** 10.1002/cbic.201900705

**Published:** 2020-04-14

**Authors:** Emrah Kara, Nis Valentin Nielsen, Bergrun Eggertsdottir, Bernd Thiede, Sandip M. Kanse, Geir Åge Løset

**Affiliations:** ^1^ Institute of Basal Medical Sciences Oslo University Hospital University of Oslo Oslo Norway; ^2^ Department of Biosciences University of Oslo 0316 Oslo Norway; ^3^ Nextera AS Oslo Norway

**Keywords:** FSAP, Marburg I, Peptide affinity tag, Phage display, Protease substrate specificity Protein−protein interactions, pVII

## Abstract

We describe a novel, easy and efficient combinatorial phage display peptide substrate‐mining method to map the substrate specificity of proteases. The peptide library is displayed on the pVII capsid of the M13 bacteriophage, which renders pIII necessary for infectivity and efficient retrieval, in an unmodified state. As capture module, the 3XFLAG was chosen due to its very high binding efficiency to anti‐FLAG mAbs and its independency of any post‐translational modification. This library was tested with Factor‐VII activating protease (WT‐FSAP) and its single‐nucleotide polymorphism variant Marburg‐I (MI)‐FSAP. The WT‐FSAP results confirmed the previously reported Arg/Lys centered FSAP cleavage site consensus as dominant, as well as reinforcing MI‐FSAP as a loss‐of‐function mutant. Surprisingly, rare substrate clones devoid of basic amino acids were also identified. Indeed one of these peptides was cleaved as free peptide, thus suggesting a broader range of WT‐FSAP substrates than previously anticipated.

## Graphical abstract

A new phage display library was constructed for protease substrate profiling in which 12‐mer combinatorial peptides are displayed in between pVII and 3XFLAG‐tag. This new library allows a simplified and streamlined protocol and testing with factor‐VII activating protease (FSAP) as bait translated into congruent results with both known, as well as novel substrate profiles.

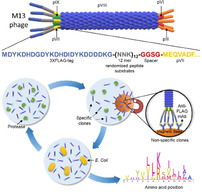

## Introduction

**Figure 1:**
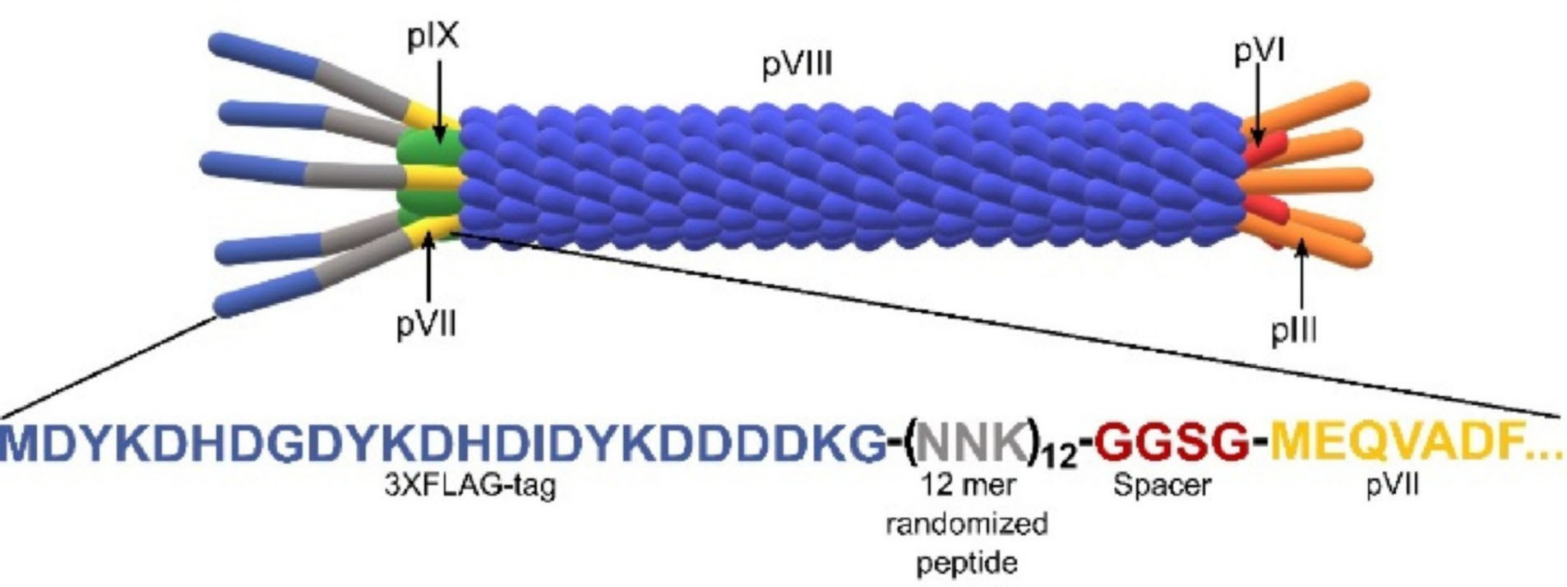
Schematic drawing of the filamentous phage structure and displayed peptides fused to pVII. NNK: Degenerate codons. N=A, T, G, C. K=T, G. Size of the proteins are not to scale.

**Figure 2:**
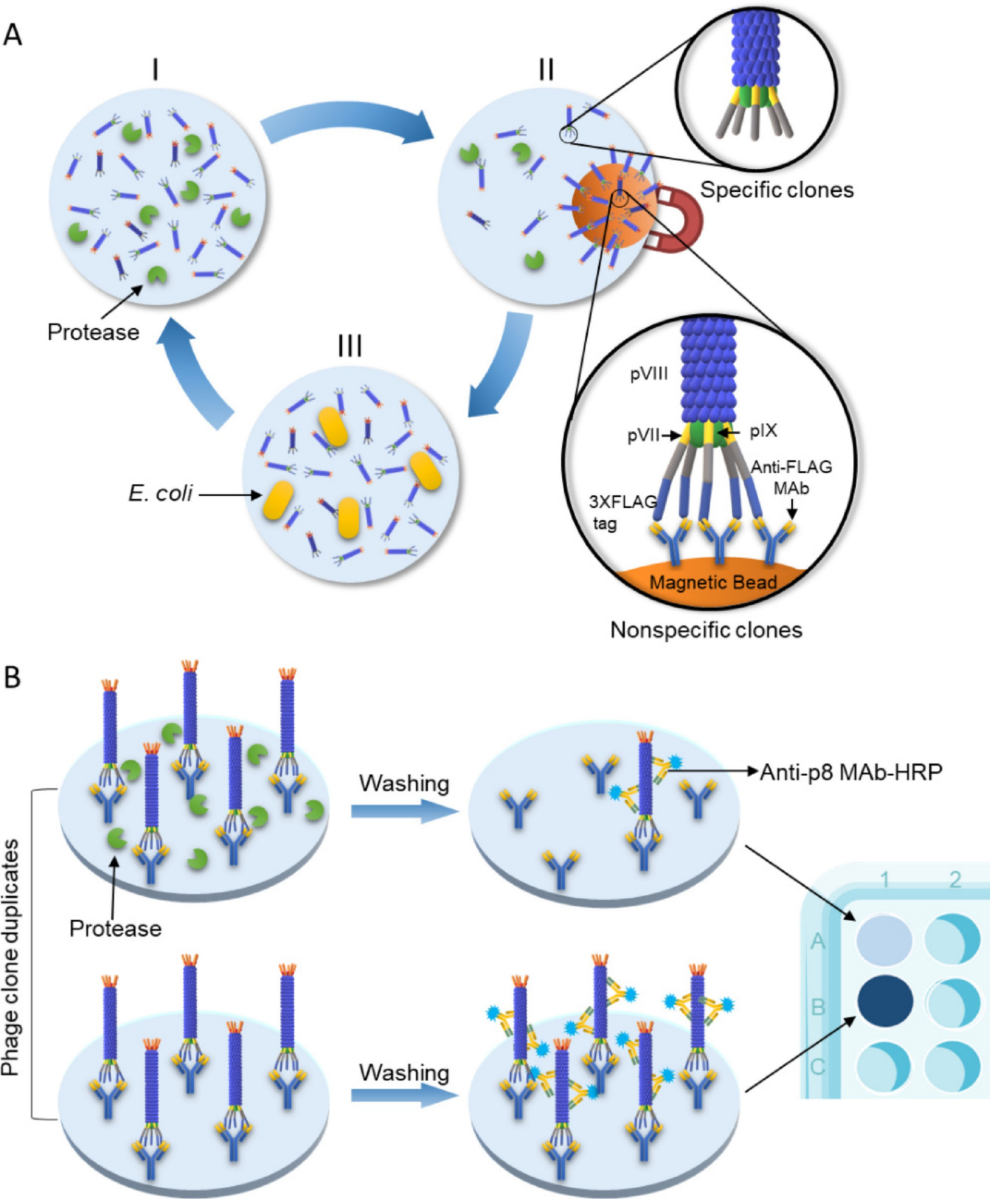
A) Phage displayed substrate selection protocol: I) Phage display library is treated with the active protease. Protease cleaves specific peptides in the library and removes their affinity tags. II) Depletion of phages displaying non‐specific peptides. Magnetic beads coupled with anti‐FLAG mAb are added to the reaction tube. Protease specific peptide substrates are cleaved and phages displaying these peptides remain in the supernatant. Non‐specific peptides with intact affinity tags, bind to magnetic beads and are separated. III) Phages with specific peptides were proliferated by infecting *E. coli*. B) Phage‐ELISA: Microtiter ELISA plate is coated with anti‐FLAG mAb to immobilize phage clones in duplicates. One of the duplicate is treated with protease, and other one receives only vector buffer without protease. The wells are washed after incubation and HRP conjugated Anti‐pVIII mAb is added to the wells and subsequently the phages are detected with HRP substrate. The difference in signal indicates specific cleavage of the peptide displayed on the phage.

The filamentous bacteriophage M13 virion consists of five different structural proteins where pIII and pVI are placed in one end of the rod‐shaped particle; pVII and pIX in the opposite end, whilst pVIII encapsulates the single‐stranded DNA (ssDNA) genome^1^ (Figure 1). Although pIII is commonly used for displaying peptides and folded proteins on the phage surface,^2^ heterologous fusion modification of pIII occasionally has shown disadvantages associated with interference in infectivity, requirement for post‐translational leader peptidase cleavage, as well as sequence restrictions in repertoires possible to display.^3^ Less is known about pVII, which is located at the opposite end of the virion. However, pVII does not rely on a leader peptide for periplasmic targeting and hence virion integration,^4^ nor does heterologous display appear to interfere with infection though differences in display properties have been reported.3a, 5 One of the applications of the phage display technology is mapping substrate specificity of the proteases.^6^ Proteases cleave specific peptides displayed in between the phage capsid protein and an affinity tag leading to loss of the tag‐; whereas phages displaying nonspecific peptides are depleted with affinity pull‐down. Specific clones in the supernatant are recovered, amplified and used for the next rounds of biopanning (Figure 2A). Following iterative rounds of selection and amplification, random clones are tested for protease‐dependent release in phage‐ELISA, where release of clones from the affinity matrix indicates *bona fide* cleavage (Figure 2B). Here, we report the construction of a novel phage displayed peptide library for protease substrate profiling where 1) 12‐mer randomized peptide substrates are fused to pVII and 2) the phage capture is mediated by a flexible and easy to handle affinity tag, namely 3X FLAG (Figure 1).

The library was tested using the recombinant protease domains of factor‐VII activating protease (FSAP) and Marburg‐I FSAP (MI‐FSAP). FSAP is a serine protease synthesized as a pro‐enzyme and secreted into the blood stream by hepatocytes.^7^ The Marburg‐I (MI) single nucleotide polymorphism (SNP) in the FSAP‐encoding gene, *HABP2,* results in a nucleotide exchange in the serine protease domain of the mature protein.^8^ MI‐FSAP has decreased protease activity against known FSAP substrates and MI‐FSAP carriers have a risk for thrombosis,^9^ stroke,^10^ carotid stenosis^11^ and liver fibrosis.^12^ It has been suggested that the low enzymatic activity of MI‐FSAP is due to incorrect transition from the zymogen to the active form.^13^ Another possibility is that the structural change caused by the single amino acid exchange in the serine protease domain of MI‐FSAP not only lowers activity, but also alters substrate specificity.

We have previously studied the substrate specificity of FSAP using both phage display and peptide scanning‐substrate combinatorial library (PS‐SCL) approaches, and observed that FSAP preferentially cleaved in regions that had clusters of basic amino acids.^14^ For those studies, we used a 7‐mer peptide phage library displayed on pIII, which relied on biotinylated AviTag as fusion capture module.^15^ The use of this library can be problematic if biotinylation is not complete, as well as pIII display may affect the infectivity, both of which would restrict the diversity of peptide pools probed.3a, 16 As an alternative approach, we here screened the new 12‐mer pVII library using the recombinant serine protease domains of WT‐FSAP (WT‐SPD) and MI‐FSAP (MI‐SPD), followed by single clone phage‐ELISA for authentication. Sequence analysis of library peptides cleaved by WT‐SPD confirmed previous findings regarding the protease specificity. A similar screen with MI‐SPD did not lead to the identification of any clones indicating that the MI‐FSAP is not likely to have an altered substrate specificity, but rather reduced enzymatic capacity. Interestingly, the WT‐FSAP screen also revealed an additional group of peptides devoid of basic amino acids as potential substrates and cleavage of one such peptide was confirmed by mass spectrometry (MS) in soluble form.

## Results

### Agarose‐gel analysis of the new phages

**Figure 3:**
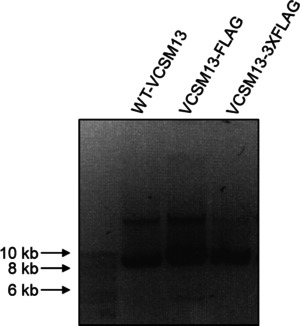
Agarose gel electrophoresis of phages particles. Equal volumes of PEG‐precipitated WT and affinity tag displaying VCSM13 phages were separated on a 1 % agarose gel, the virions were denatured, and the ssDNA content was visualized as described in the experimental section.

The phage genome is sensitive to genetic alternations, which might translate to altered phenotype, such as multiunit length polyphages, adversely affecting downstream applications.^17^ The pVII modified and control phages were therefore characterized by agarose gel analysis to assess for normal morphology,^17^, ^18^ and the results showed expected and similar unit sizes of all species (Figure 3). To facilitate library generation, a stuffer was introduced into VCSM13‐3XFLAG tag genome, which contained consecutive Amber (TAG) stop codons to minimizing template background in the final library when produced in a non‐suppressor host.

### Phage affinity‐matrix binding assays

**Figure 4:**
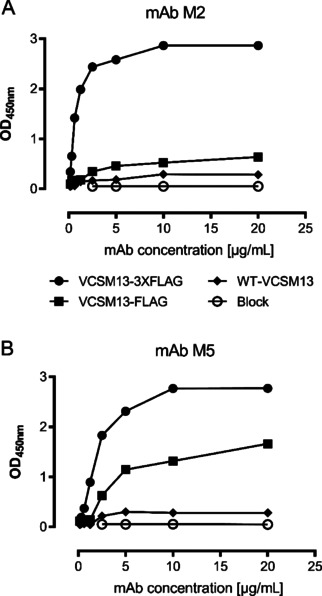
Phage capture efficiencies of phages. Constant amount of phages; VCSM13, VCSM13‐FLAG or VCSM13‐3xFLAG in phage capture ELISA with serial dilutions of antibodies. A) Anti‐FLAG mAb M2. B) Anti‐FLAG mAb M5. The experiment was performed in duplicate and the mean is shown.

A phage capture ELISA was performed using two different anti‐FLAG mAbs, M2 and M5 to assess the affinity‐matrix binding performance of the phages VCSM13‐FLAG and VCSM13‐3XFLAG. The M13 phage detection with a polyclonal Ab directed against pVIII was included as a positive control for virion presence. VCSM13‐3XFLAG performed better compared to VCSM13‐FLAG in binding to affinity matrixes coated with either M2 (Figure 4A) or M5 antibody (Figure 4B).

### Phage‐displayed substrate library generation

**Figure 5:**
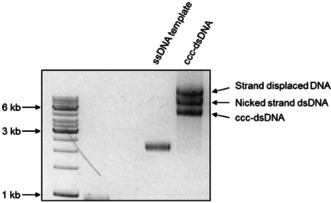
In vitro synthesis of ssDNA to ccc‐dsDNA analyzed on the agarose gel. DNA samples were run in 2 % agarose gel and visualized by GelRed nucleic acid stain.

A peptide substrate library was generated by fusing 12‐mer randomized sequences with *N*‐terminal 3XFLAG tag to coat protein pVII of the M13 phage essentially as described^19^ (Figure 1). Briefly, the stuffer‐modified phage was produced in *E. coli* XL1‐Blue amber suppressor cells, ssDNA isolated and converted into covalently‐closed‐circular double‐stranded DNA (ccc‐dsDNA) using appropriate library oligos (Figure 5). ccc‐dsDNA was electroporated into amber non‐suppressor *E. coli* SS320 bacteria obtaining 2.9×10^8^ primary transformants with ∼40 % recombination efficiency, resulting in a library diversity of about 1.2×10^8^ unique clones.

### Phage displayed substrate selection

Three rounds of consecutive biopanning was performed using WT‐SPD and MI‐SPD as target proteases for selecting specific substrates. Briefly, the library aliquots were treated with WT‐SPD and MI‐SPD separately at 37 °C for one hour (h), in parallel with untreated library aliquots as base line control. Uncleaved phages retaining their affinity tag were then depleted using magnetic beads coated with anti‐FLAG M2 antibodies, and the remaining phages were rescued by *E. coli* infection and amplified for further selection or analysis.

### Phage‐ELISA

**Figure 6:**
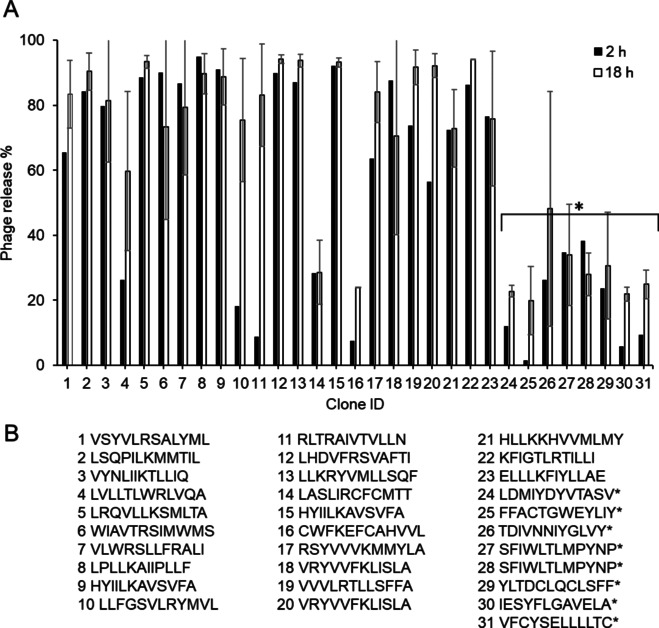
Identification of phage clones cleaved by FSAP. A) Phage‐ELISA; Individual clones were selected randomly and treated with WT‐SPD for 2 and 18 hours. After washing steps, phages were detected by anti‐M13‐HRP mAb as absorbance 405 nm. Results are shown for 31 clones that showed a greater than 20 % cleavage by WT‐SPD compared to buffer control. These results represent 1 experiment performed in singlet for 2 h and 2 independent experiments performed in singlet for 18 h (mean+SD, duplicates). B) Peptide sequences of the variable regions displayed on the phage. * Clones do not contain Arg or Lys.

We did a time‐dependent parallel screening of 360 individual phage clones from the third panning round of WT‐SPD and MI‐SPD selection by phage‐ELISA. There was no significant phage release in the MI‐SPD screen with either 2 h or 18 h incubation (data not shown). In contrast, 24 individual phage clones were significantly released (threshold set to 20 % as compared to untreated) in the presence of WT‐SPD in the ELISA after 2 h incubation and this number raised to 31 after 18 h incubation (Figure 6A). All clones were sequenced and split into two distinct groups either containing basic amino acids in their putative FASP substrate sequences, or not (Figure 6B). The low general release rate in phage‐ELISA even after 18 h also largely confirms the 3X FLAG tag to be inert to FSAP activity.

### Cleavage of synthetic peptides based on the clones selected

**Figure 7:**
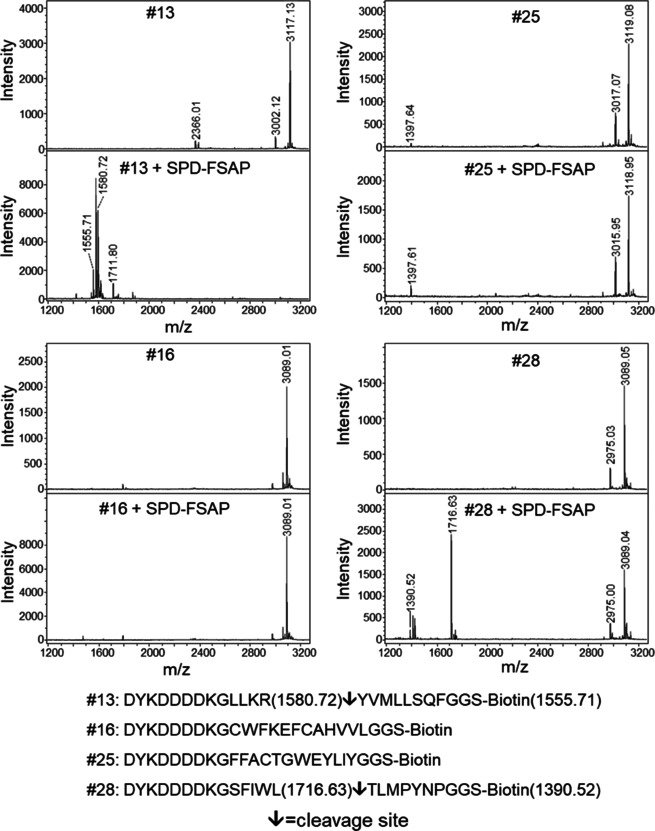
Cleavage of synthetic peptides #13, #16, #25 and #28 by WT‐SPD. MALDI‐MS spectra of untreated peptides (control) or incubated with WT‐SPD (10 μg/mL) for 2 h at 37 °C. Sequence and the positions of cleavage are indicated by arrows.

**Figure 8:**
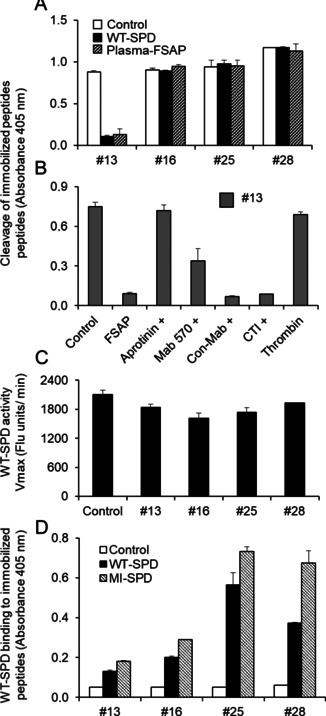
Characterization synthetic FSAP substrate candidate peptides. A) Biotinylated peptides (2 nM) were captured on neutravidin‐coated wells (2 μg/mL). Recombinant WT‐SPD or plasma FSAP (5 μg/mL) was added to the wells for 2 h at 37 °C. Residual peptide levels remaining on the plate were detected with anti‐FLAG‐HRP. B) As above except that the cleavage of peptide #13 with plasma FSAP was measured in the presence of aprotinin (50 μg/mL) mAb570, control mAb (both at 25 μg/mL) and corn trypsin inhibitor (CTI) (40 μg/mL). Human thrombin (Sigma Aldrich) was tested at a concentration of 0.5 U/mL. The amount of biotinylated peptide remaining on the plate is presented as absorbance at 405 nm (mean+SD, duplicates). C) Recombinant WT‐SPD (2 μg/mL) was pre‐incubated with peptides (50 μM) for 30 min before measurement of fluorescent substrate hydrolysis (Vmax, fluorescence units/min; mean+SD, duplicates). D) Biotinylated peptides (5 μM) were captured on neutravidin‐coated wells (2 μg/mL). Recombinant WT or MI‐SPD (2 μg/mL) was added to the wells for 1 h at 37 °C and binding was detected with an anti‐FSAP antibody as absorbance at 405 nm (mean+SD, duplicates).

The phage‐ELISA revealed some surprising results, including cleavage of peptide sequences without basic amino acids, and discrepancy between 2 h and 18 h incubations. In phage‐ELISA, we expected increased phage release rate after 18 h incubation compared to 2 h for the same phage clones; however, additional clones were found after 18 h indicating potentially unspecific release at the longest protease exposure. To shed light on possible specificity differences in this regard, we tested four peptides of the selected phage clones for the cleavage specificity in the form of soluble peptides using MALDI‐TOF‐ mass spectrometry (MS) and ELISA. Clones were selected based on release in phage‐ELISA for 2 h and 18 h incubation, as well as presence or absence of basic amino acids. Clone 13 and 28 were released well after 2 h, whereas clones 16 and 25 were released only after 18 h incubation. Further, 13 and 16 both contained the anticipated basic amino acids (Lys/Arg) whereas 25 and 28 did not. The synthetic peptides were made with an *N*‐terminal FLAG and a *C*‐terminal biotin tag to mimic both the phage context from which they were identified, as well as facilitate capture and detection in ELISA (Figure 7). In MS analysis, peptides found in 2 h incubation #13 and #28 were cleaved by FSAP, whilst peptides found in 18 h incubation #16 and #25 were not. Scissile bond was found after Arg for #13 as expected, for #28 scissile bond was between Leu and Thr (Figure 7). For all of the peptides tested in MS, FLAG‐tag remained unaffected by FSAP activity. To independently validate the MS result, we also investigated cleavage by ELISA. Here, both recombinant WT‐SPD and plasma FSAP cleaved peptide #13, whereas none of the other peptides were cleaved (Figure 8A). The cleavage of peptide #13 by plasma FSAP was partially blocked by an inhibitory monoclonal antibody to FSAP (mAb570), but not with a control antibody. Similarly, the known FSAP inhibitor aprotinin strongly affected peptide #13 cleavage, whereas this was not seen with the no‐FSAP protease inhibitor corn trypsin inhibitor (CTI). A related protease, thrombin, did neither not cleave peptide #13 (Figure 8B). None of the peptides had any modulatory effect on the enzymatic activity of WT‐SPD (Figure 8C). The binding of the four peptides to immobilized WT‐SPD and MI‐SPD was tested. The peptides without any basic amino acids, #25 and #28, bound more strongly as would be expected of hydrophobic peptides compared to hydrophilic peptides. Binding to MI‐SPD was higher than that to WT‐SPD, but there was no pattern between binding ability and cleavage by the protease (Figure 8D).

## Discussion

We here report, to the best of our knowledge, the first phage‐displayed protease substrate library that uses the filamentous phage capsid pVII as display scaffold. Although pIII is widely used for phage display, pVII has some potential advantages in that pVII display does not affect infectivity,3b as well as does not rely on the leader peptide‐dependent SEC translocon for periplasmic targeting that may differentially affect the identity of the peptide pool available for probing.3a

The affinity capture tag is an important component of the substrate phage display system to immobilize the randomized peptide library. The selected tag should be homogeneously present on all the phages of the library, and the binding to its affinity matrix should be strong, specific and durable. In our previous study of FSAP substrate specificity, 18‐mer *E. coli* biotin ligase (BirA) recognition sequence (AviTag) was employed as the capture module of the phage display substrate library.^14^ Although this tag is readily compatible with pVII display and biotinylated phages bind to avidin matrices strongly, *in vivo* biotinylation process introduces additional processing steps during phage packaging, as well as biotinylation levels varies and are difficult to consistently get to full efficiency.^20^

3XFLAG‐tag, a 22 amino acid long peptide is consisting of three tandem FLAG tags. It is highly hydrophilic and with a negative net charge well compatible with the filamentous virion, and it binds strongly with high specificity to commercially available anti‐FLAG antibodies.^21^ We compared the binding capacity of FLAG and 3XFLAG phages using two different anti‐FLAG mAbs, namely M2 and M5 (Figure 4). 3XFLAG performed better than FLAG in binding to both Abs. Thus, 3XFLAG‐tag was chosen for the library construction. Furthermore, we have included a short spacer (Gly−Gly−Ser−Gly) in between substrate peptide and pVII to give enough flexibility for the substrate peptide to interact with protease (Figure 1).

The new library was tested with SPD of WT‐ and MI‐FSAP. With the phage outputs from MI‐SPD and WT‐SPD library screenings, we performed phage‐ELISA assay for identifying positive clones displaying specific substrate peptides. Duplicate samples of randomly selected clones were incubated with the proteases for either 18 h as previously reported,^14^ or for 2 h to see if we could condense the protocol without compromising efficiency. Indeed, positive clones were identified in the WT‐SPD screen, and more clones found positive in 18 h compared to 2 h (Figure 6A). The majority of the substrate peptide candidates contained at least one basic amino acid, thus complementing our previous observations. Surprisingly, a distinct group of substrate peptide candidates were devoid of basic amino acids (Figure 6B). The MI‐SPD screen did not identify any clones exhibiting significant phage release, hence supporting the hypothesis that MI‐FSAP is a variant with lower protease activity.

Studies with synthetic peptides were performed to further elucidate these substrate peptide candidates as potential FSAP substrates using ELISA and MS. To investigate putative specificity differences in obtained clones dependent on the two digestion time intervals used for screening, we selected clones 13 and 28 that were released after 2 h incubation in phage‐ELISA, and clones 16 and 25 that were released only after 18 h incubation. Further, clones 13 and 16 contained basic amino acids, while 25 and 28 did not. Indeed, the MS experiment showed that only, peptides #13 and #28 were cleaved by FSAP, thus pointing to peptides #16 and #25 being false positives being spontaneously released as course of the long incubation.

Three of the sequences identified in the screen by phage‐ELISA for 2 h incubation did not contain basic amino acids; nonetheless, the MS analysis showed that peptide #28, devoid of basic amino acids, was reproducibly cleaved after the hydrophobic amino acid Leu. This observation does not fit well with the fact that there is an aspartate in the S1 pocket of FSAP, which supposedly determines the preference for basic amino acids for efficient cleavage.^14^

Notably, the release percentage of the clones without Arg or Lys was lower than clones containing Arg or Lys (Figure 6A), which may indicate that they are weaker substrates for FSAP. Indeed, the cleavage of the peptide #28 lacking basic amino acids was confirmed by MS; however, the cleavage was not detectable by ELISA. On the other hand, cleavage of the peptide #13 with basic amino acids was detected in both experiments. Higher detection sensitivity of MS compared with ELISA may partially explain the discordance between the experiments, but may also indicate less suitable substrate peptides to FSAP.

**Figure 9:**
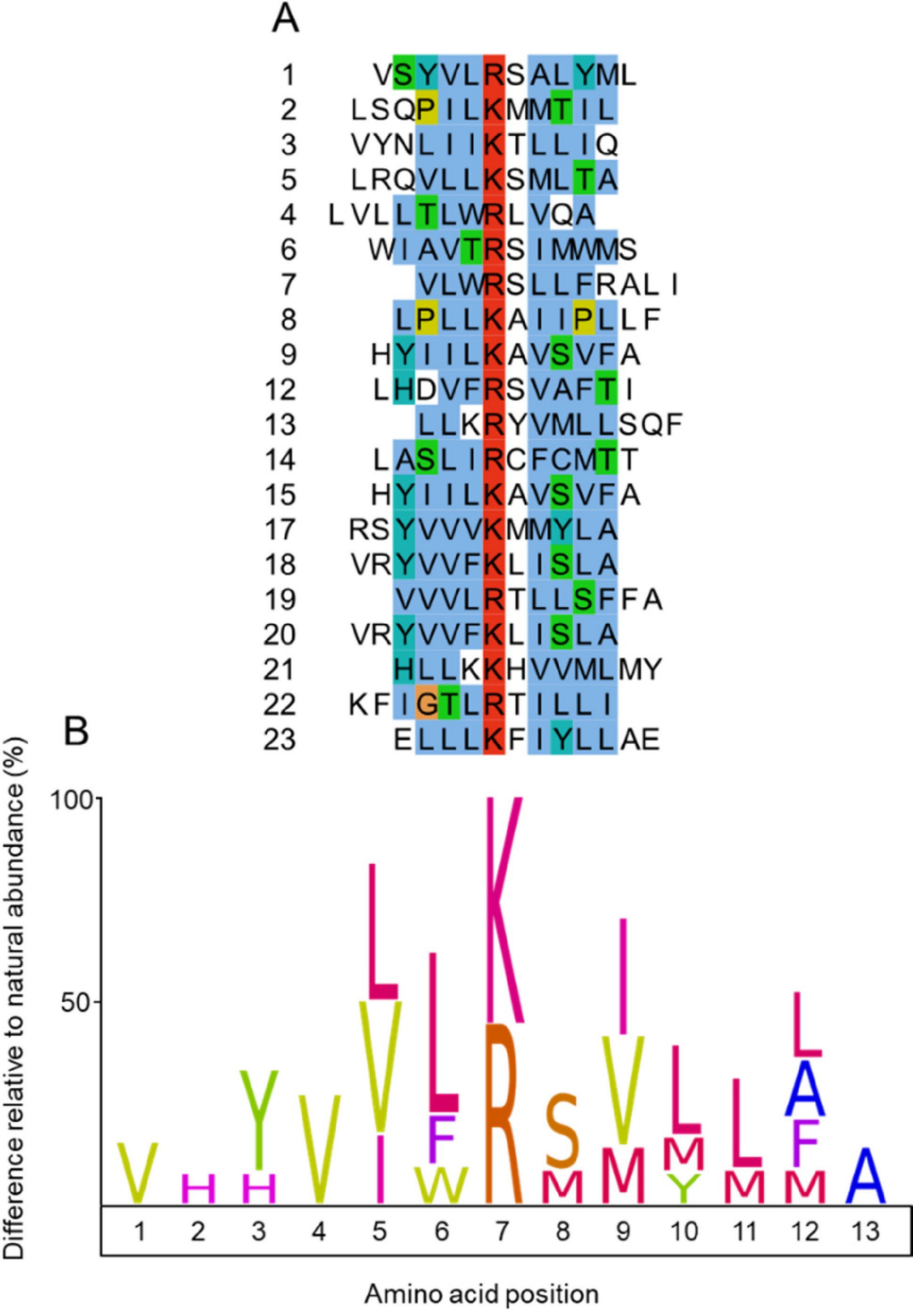
Analysis of the WT‐SPD specific sequences identified by phage display. Phage clones cleaved by WT‐SPD after 2 h incubation were sequenced. A) The peptide sequences contained basic amino acids aligned manually using online software JABALS 2.1 and colored by the ClustalX color scheme. B) The fold enrichment of the amino acids in each position is represented as an IceLogo. Clone numbers are same as the Figure 6.

Positive clones identified in the WT‐SPD screen had basic amino acids and thus showed similarity to our previous data; most of the peptide sequences had a basic amino acid in between small polar and nonpolar amino acids (Figure 9A). The alignment of these sequences gave a consensus Leu−Arg/Lys−Ser−Ile/Val/Met which is close to our previous result Leu−Lys−Arg−Ser−Val^14^ (Figure 9B). In our previous study, all of the peptide substrates had at least one basic amino acid. Moreover, the majority of sequences, and accordingly the consensus sequence, had two tandem basic amino acids at the cleavage site indicating enrichment of substrate peptides with uniformity and similar affinity.^14^ In the current study, only two of the 2 h substrate sequences had two tandem basic amino acids, whereas most of the substrate sequences have one basic amino acid at the cleavage site (Figure 9A). In addition, we found the sequences devoid of basic amino acids altogether. Thus, it appears as if the current result covers a wider substrate specificity pool than in the past assessment. Indeed, we noted that one of the cleavage sites for FSAP in fibrinogen alpha chain was also after a non‐basic amino acid in an earlier study.^22^ Our current observations, thus support further studies to understand the nature of the cleavage at non‐basic amino acids and the search for alternative yet unknown substrates for FSAP.

## Conclusion

In the current study we have demonstrated the applicability of pVII as an alternative phage display library scaffold for identifying protease specificity exemplified by FSAP. The previous observation of a FSAP substrate consensus motif centered on basic amino acids was confirmed using a simplified and faster protocol. In addition, we made the novel observation that FSAP can cleave a peptide without any basic amino acid. Further, our data support the notion that the MI variant of FSAP is a loss‐of‐function mutant with very low catalytic capacity. In summary, our new library provides an easy to handle and useful addition to the substrate library approach for the study of protease specificity using phage display.

## Experimental Section:

**Construction of phage with affinity tags**: VCSM13 (GenBank accession no.: AY598820) helper phage was purchased from New England Biolabs (Ipswich, MA, USA). A DNA fragment encoding the 3XFLAG (MDYKDHDGDYKDHDIDYKDDDDK) fused to the pVII N‐terminus was obtained in pUC57 vectors from GenScript Inc (NJ, USA). The insert was subcloned into the VCSM13 backbone using the unique *BsrGI/SnaBI* RE site as previously described.20a In‐house prepared electrocompetent *E. coli* XL1‐Blue cells (recA1 endA1 gyrA96 thi‐1 hsdR17 supE44 relA1 lac [F′ proAB lacIqZΔM15 Tn10 (Tetr)]) were transformed with modified VCSM13 DNA. *E. coli* XL1‐Blue harboring the VCSM13 DNA were grown in 2x YT media (Yeast Extract, Tryptone, NaCl, Sigma‐Aldrich, Oslo, Norway) with 50 μg/mL kanamycin (Sigma‐Aldrich) at 37 °C for 18 h with 200 rpm shaking. Supernatant were separated from cell with centrifugation at 400 rpm 30 minutes and the phages in the supernatant were concentrated by PEG/NaCl purification as described before.20a

**Agarose gel analysis of the newly constructed phages**: The newly constructed helper phage particles were analyzed as described by Mount et al.^18^ 20 μL of phage solution was run in the 1 % agarose gel. The gel was then incubated in 100 mL of 0.2 M NaOH for 1 h with agitation and neutralized with 1 M Tris−HCl, pH 7.0 after washing with H_2_O. Agarose gel was incubated with GelRed™ nucleic acid gel stain (Biotium, Hayward, USA) for 1 h and visualized with UV light source. To compare new phages, WT‐VCSM13 was used as a control.

**Phage affinity‐matrix binding assays**: 96 well microtiter plates (Costar 96 Well EIA/RIA Plate (Corning Incorporated, Corning, NY, USA)) were coated with 5 μg/mL of anti‐FLAG M2 and M5 (Sigma Aldrich, Oslo, Norway) in 1x PBS (phosphate buffered saline) at room temperature (RT) for 1 h. The plate was blocked with PBST‐SMP (PBS supplemented with 0.01 % (v/v) Tween 20 and 4 % (w/v) skimmed milk powder (SMP)). The plates were then incubated for 3 h/RT. 100 μL of sample (either 10^10^ or 10^11^ cfu^cam^/mL or 10‐fold dilution series) was added to each well and incubated for 1.5 h/RT. The bound phages were detected with 100 μL anti‐M13‐HRP (1 : 3000) in PBST for 1.5 h/RT. The wells were developed with 100 μL TMB soluble substrate (Calbiochem, Darmstadt, Germany) by the addition of 100 μL/well. The reaction was stopped after 5 min by adding 100 μL/well of 1 M HCL. The absorbance was measured at A450 nm using a TECAN ELISA reader apparatus. Between each step, the wells were washed 3x with PBST (300 μL/well) using the TECAN ELISA washer.

**DNA template construction for phage‐displayed peptide substrate library generation**: A 12‐mer randomized phage‐displayed peptide substrate library was designed based on the principles described by Scholle et al. with modifications^24^. The library was constructed using a modified protocol of Tonikian et al..^19^ Briefly, *E. coli* strain XL‐1 Blue was infected by the VCSM13‐3XFLAG phage and grown in 2x YT supplemented with 50 μg/mL kanamycin at 37 °C for 18 h. Cleared supernatant was used as source for isolation of phage double stranded DNA (dsDNA) isolated with NucleoSpin® Plasmid kit (Macherey‐Nagel, Durel, Germany). A 52 nucleotide long stuffer‐sequence was inserted in to the phage genome between DNA sequences encoding 3XFLAG‐tag and pVII protein with Q5 site directed mutagenesis kit (New England Biolabs) with the mutagenic primers 12aaSTF.For and 12aaSTF.Rev (See Table 1 for all oligonucleotide sequences). Forward and reverse primers were designed according to instructions of the kit manufacturer. Chemically competent XL‐1 Blue cells were transformed with *in vitro* synthesized double‐stranded mutagenic phage DNA and plated on LB Agar (Sigma‐Aldrich) supplemented with 50 μg/mL kanamycin. Kanamycin resistant colonies were picked and expanded for sequence verification and phenotypic assessment.

**Table 1:** List of oligo‐nucleotides.

Name	Sequence
12aaSTF.For	5′‐CCTGTATTTCCAGGGCGGTAGCGGTATGGAGCAGGTCGCGGAT‐3′
12aaSTF.Rev	5′‐TTCTCGCGGCCGCTCTAGCTCTAGCCTTTATCATCATCATCTTTATAATCAATATCATGATCTTTATAATC‐3′
pVII_for	5′‐AGCAGCTTTGTTACGTTGATTTGG‐3′
pVII_rev	5′‐GCAGCGAAAGACAGCATCG‐3′
Randomized library oligos^[a]^	5′‐CATACCGCTACCGCCMNNMNNMNNMNNMNNMNNMNNMNNMNNMNNMNNMNNGCCTTTATCATCATC‐3′

[a] Reverse complement (MNN: Reverse complement of NNK codon)

**Phage detection by M13 enzyme‐linked immunosorbent assay (ELISA)**: The phage production in the bacterial cultures was screened by phage detection enzyme‐linked immunosorbent assay (ELISA). Maxi‐Sorp™ microtitre plate wells (Nunc, Roskilde, Denmark) were coated with 50 μL of 5 μg/mL anti‐M13 antibody (GE Healthcare, Uppsala, Sweden) in PBS at 4 °C for 18 h. The wells were blocked with 100 μL PBST‐SMP, for 1 h at 22 °C. Supernatants of the cultured colonies were added to wells and incubated for 1 h at 22 °C. The plate was washed and the phages were detected with anti‐M13‐HRP (GE Healthcare, Uppsala, Sweden) (1 : 5000 dilution in PBST/BSA (PBS supplemented with 0.01 % (v/v) Tween 20 and 3 % (w/v) BSA). The insertion of the stuffer was verified by Sanger sequencing with primers pVII‐for and pVII‐rev (Table 1). The phage titration was determined by optical density (OD) as particle/mL (OD 1.0 at 268 nm of a solution contain 5×10^12^ phage/mL), and by infectious titration as infective phage/mL.^24^

**Phage‐displayed substrate library generation**: Bacteria expressing phages with verified stuffer sequence were propagated and the supernatant was collected and the phages precipitated with PEG/NaCl as described before.20a ssDNA was isolated with QIAprep Spin M13 Kit (Qiagen Nordic, Oslo, Norway). ssDNA was converted into ccc‐dsDNA with synthetic randomized library oligos (Table 1) and electroporated in to elecrocompotent *E. coli* strain SS320 cells (MC1061F\′) (Lucigen, WI, USA). Number of transformants was determined by plating 1 μL cells on kanamycin supplemented LB agar, and counting kanamycin resistant colonies. 20 colonies were randomly selected from kanamycin resistant colonies and grown overnight separately in 5 μL 2x YT+kan. Phage dsDNA was isolated with NucleoSpin plasmid kit and DNA sequenced and transformation efficiency was identified. After transformation, bacteria have grown in 2x YT supplemented with 50 μg/mL kanamycin and phages in the supernatant were precipitated and concentrated with PEG/NaCl described above. Library diversity was calculated with the formula: diversity=(number of transformants)×(recombination efficiency (%)).

**Phage‐displayed substrate selection**: Recombinant serine protease domain WT (WT‐SPD) and MI‐SPD were prepared and activated as described before.^25^ The library containing 1×10^10^ phages was blocked with PBST/BSA for 1 h at 22 °C. Protease (30 nM) or buffer control was added into separate phage library aliquots and incubated 1 h at 37 °C. After incubation, an FSAP inhibitor aprotinin (90 nM) was added.^26^ Anti‐FLAG M2 Magnetic Beads were blocked by PBST/SMP for 1 h at 22 °C and washed with PBST. Protease and control treated phage display library aliquots were incubated with magnetic bead aliquots separately for 1 h at 22 °C and after magnetic separation of the beads; each supernatant was incubated with another aliquot of fresh magnetic bead at the same conditions as before and supernatant was separated. *E. coli* strain XL1‐Blue cells were infected by the phages in the supernatant; cells were grown and phages were produced at 30 °C for 18 h with shaking at 200 rpm. Supernatant were separated from cell with centrifugation at 400 rpm for 30 minutes and the phages in the supernatant were concentrated by PEG/NaCl purification and used for the next round of bio‐panning under the same conditions.

**Phage enzyme‐linked immunosorbent assays (Phage‐ELISA)**: After third panning round, phage substrate ELISA was performed as described before.^14^, ^19^ Individual phage clones were randomly picked from the third panning round out‐put of the both MI‐SPD and WT‐SPD and amplified as described before.20a Maxi‐Sorp™ microtitre plate wells were coated with 50 μL of 5 μg/mL M5 Anti‐FLAG ab in PBS at 4 °C for 18 h. The wells were blocked with 100 μL PBST/SMP for 1 h at 22 °C. Amplified phage clone supernatants were added to wells in duplicates and incubated for 1 h at 22 °C. After washing three times with TBST, one of the wells was treated with 50 μL, 30 nM protease in reaction buffer PBST and the other well of the duplicate was treated with reaction buffer only without protease and incubated at 37 °C for 2 h and 18 h separately. The plate was washed and the phages were detected with anti‐M13‐HRP (1 : 5000 dilution in PBST/BSA). The signal was developed with 1‐Step Ultra TMB ELISA Substrate (ThermoFisher Scientific, Oslo, Norway), The relative phage release for each phage clone was determined as the ratio of signals from reactions with FSAP compared to control buffer. Phage clones with 20 % or more difference in phage detection signal were DNA sequenced. 20 % was decided as an arbitrary cutoff point. Primers pVII‐frwd and pVII‐rev were used in PCR reactions with phage template from an aliquot of phage culture to amplify the M13 phage pVII gene region with the randomized peptide library. Peptide sequences were aligned manually performed online software JABALS 2.1,^27^ colored by ClustalX default coloring scheme,^28^ and represented by IceLogo.^29^

**Cleavage of immobilized biotinylated peptides by FSAP**: Synthetic peptides based on the sequence of the clones 13, 16, 25 and 28 were synthesized with a N‐terminal FLAG‐tag and C‐terminal biotin (Genscript) and peptides were depicted as #13, #16, #25 and #28 (Figure 7). ELISA plates were coated with neutravidin (2 μg/mL). After blocking with BSA (3 % w/v) peptides (2 nM) were captured. Wells were treated with plasma‐FSAP or WT‐SPD in the absence or presence of inhibitors in a buffer with 25 mM Tris (pH 7.4), 137 mM NaCl, with 2 mM CaCl_2_, 0.3 % (w/v) BSA and 0.1 % (w/v) Tween‐20. After incubation for 2 h at 37 °C the plates were washed extensively and developed with peroxidase coupled anti‐FLAG antibody and developed with TMB substrate.

**Peptide cleavage analysis by mass spectrometry**: Peptides (200 μM) were incubated with WT‐SPD at 37 °C for 2 h in TBS (50 mM Tris, pH 7.4, 100 mM NaCl, Tween‐20 (0.1 % w/v) and CaCl_2_ (2 mM). Reactions were stopped with final concentration of trifluoroacetic acid (TFA) (0.2 % v/v). The peptides were analysed by matrix‐assisted laser desorption/ionization time‐of‐flight mass spectrometry (MALDI‐TOF‐MS) in the reflectron mode using an Ultraflex II (Bruker Daltonics, Bremen, Germany). The peptides were purified using μ‐C18 ZipTips (Millipore, Billerica, MA, USA) and directly spotted onto the MALDI target with 0.6 μL matrix (20 mg/mL α‐cyano‐4‐hydroxycinnamic acid in 0.25 % aqueous TFA/acetonitrile (2 : 1)). MS spectra were evaluated using the software FlexAnalysis version 2.4. (Bruker Daltonics).

**Binding of WT‐SPD to immobilized peptides**: After capturing peptides (5 μM) on plates, as described above, WT‐SPD (2 μg/mL) was added to the wells and its binding detected with anti‐FSAP antibody.

**Effect of peptides on the enzymatic activity of WT‐SPD**: Hydrolysis of a fluorescent substrate was measured using a Synergy HI plate reader (BioTek Instruments, Winooski, USA) with excitation at 320 nm and emission at 460 nm (37 °C for 1 h) as described before.^14^ The initial velocity was calculated from the initial linear part of the progress curve. The standard assay system consisted of TBS (25 mM Tris−HCl, pH 7.5, and 150 mM NaCl) with CaCl_2_ (2 mM) and Tween‐20 (0.1 % w/v). Peptides (50 μM) were pre‐incubated with WT‐SPD (2 μg/mL) before starting the enzyme assay.

## Conflict of interest

G.Å.L. is inventor on a patent describing pVII phage display. All other authors disclose no conflict of interest.

## Supporting information

As a service to our authors and readers, this journal provides supporting information supplied by the authors. Such materials are peer reviewed and may be re‐organized for online delivery, but are not copy‐edited or typeset. Technical support issues arising from supporting information (other than missing files) should be addressed to the authors.

Supplementary
