# Supplementary material for: Design and Characterization of a New pVII Combinatorial Phage Display Peptide Library for Protease Substrate Mining Using Factor VII Activating Protease (FSAP) as Model
Source: Chembiochem. 2020 Apr 14;21(13):1875–84. doi: 10.1002/cbic.201900705 (PMC7383712; doi:10.1002/cbic.201900705)
Supplement: Supplementary file 1 — Supplementary [file CBIC-21-1875-s001.pdf]

Author Contributions

E.K. Data curation:Lead; Formal analysis:Lead; Methodology:Lead; Validation:Lead; Visualization:Lead; Writing - Original Draft:Lead; Writing - Review & Editing:Lead
N.N. Methodology:Equal; Resources:Equal; Writing - Review & Editing:Supporting
B.E. Investigation:Supporting; Methodology:Equal; Writing - Review & Editing:Supporting
B.T. Formal analysis:Supporting; Investigation:Equal; Methodology:Equal; Writing - Review & Editing:Supporting
S.K. Conceptualization:Lead; Funding acquisition:Lead; Project administration:Equal; Supervision:Lead; Writing - Original Draft:Lead; Writing - Review & Editing:Lead
G.L. Conceptualization:Lead; Formal analysis:Lead; Funding acquisition:Lead; Investigation:Lead; Project administration:Lead; Supervision:Lead; Writing - Original Draft:Supporting; Writing - Review & Editing:Lead
